# Experimental evidence of early-phase transmission of *Yersinia pestis* by *Synopsyllus fonquerniei* and *Xenopsylla brasiliensis*

**DOI:** 10.1371/journal.pntd.0014463

**Published:** 2026-06-30

**Authors:** Ravo Rakotobe Harimanana, Florent Sebbane, Mireille Harimalala

**Affiliations:** 1 Medical Entomology Unit, Institut Pasteur de Madagascar, Antananarivo, Madagascar; 2 Ecole Doctorale Sciences de la Vie et de l’Environnement (SVE), Université d’Antananarivo, Antananarivo, Madagascar; 3 Univ. Lille, CNRS, Inserm, CHU Lille, Institut Pasteur de Lille, U1019 – UMR – CIIL – Center of Infection and Immunity of Lille, Lille, France; Lanzhou University, CHINA

## Abstract

**Background:**

Flea-borne transmission of the plague bacillus, *Yersinia pestis*, occurs through two regurgitation-based mechanisms. One, called early-phase transmission (EPT), takes place within 24–96 hours post-infection through a poorly understood mechanism. The second requires an intrinsic incubation period and relies on the formation of a biofilm obstructing the flea’s proventriculus. Although these mechanisms have been described in different flea species, they have never been characterized in *Xenopsylla brasiliensis* and *Synopsyllus fonquerniei*, which are two flea vectors suspected and confirmed to contribute to *Y. pestis* transmission in Madagascar. Because EPT may facilitate rapid pathogen spread, this study aimed to experimentally assess the ability of both flea species to transmit *Y. pestis* via EPT.

**Methodology:**

Cohorts of starved *X. brasiliensis* and *S. fonquerniei* fleas were artificially infected with *Y. pestis*. From day 1 (D1) to day 4 (D4) post-infection (p.i.), pools of ten infected fleas were fed on sterile mouse blood. After feeding, bacterial loads in both the blood and the fleas were determined by Colony Forming Unit (CFU) counts on blood agar to assess bacterial transmission. Fleas were also observed under a binocular microscope to determine whether the foregut was blocked, as indicated by the presence of fresh blood in the foregut but not in the midgut.

**Principal findings:**

The results showed that *X. brasiliensis* and *S. fonquerniei* were able to transmit *Y. pestis* as early as 24 hours p.i., although transmission events were infrequent. No correlation was found between flea bacterial load (ranging from 4.0 x 10^1^ to 4.4 x 10^5^ CFU) and transmission success. Blockage was observed in 1% of *S. fonquerniei* (D3 and 4 p.i) and 0.75% of *X. brasiliensis* (D4 p.i.), without detectable transmission by these blocked fleas during the experimental window.

**Conclusion:**

This study provides the first experimental evidence that *X. brasiliensis* and *S. fonquerniei* can support early-phase transmission of *Y. pestis* and are capable of developing proventricular blockage under laboratory conditions. These findings refine our understanding of the potential contribution of these flea species to plague transmission dynamics in Madagascar.

## Author summary

Plague is a zoonotic disease caused by the bacterium *Yersinia pestis* and transmitted to humans and animals through flea bites. Fleas can transmit the bacterium in two mechanisms: early-phase transmission, which occurs rapidly after infectious bloodmeal, and proventricular blockage, which develops more slowly. For *Xenopsylla brasiliensis* and *Synopsyllus* *fonquerniei* in Madagascar, these transmission mechanisms had not yet been characterized. In our laboratory experiments, we found that both species were able to transmit the bacterium via early-phase transmission, although this occurred infrequently. They were also able to develop proventricular blockage within a few days, but blocked fleas did not transmit bacteria during our experiments. These results provide the first experimental evidence that these flea species can support early-phase transmission and improve our understanding on how plague may spread in Madagascar. These findings highlight the importance of including these flea species in plague surveillance and vector management strategies.

## Introduction

Plague is a fatal disease that remains a serious public health concern in some countries of the world, particularly in Madagascar [1]. It is caused by the bacterium *Yersinia pestis* and is transmitted by fleas. Flea-borne transmission of *Y. pestis* occurs through two regurgitation-based mechanisms. One, called early-phase transmission (EPT), takes place within 24–96 hours following flea infection and fades away after subsequent uninfected blood meals [2]. The biological basis of this transmission remains unclear, although it has been suggested that EPT requires a high level of bacteremia in the infected blood [3,4]. The bacterial load transmitted during the early phase is thought to be very low, and transmission can occur either *en masse* or individually [4,5]. The second mode of flea-borne transmission requires an intrinsic incubation period and may occur between 5 days and three weeks post-infection [2,6]. It relies on the formation of a thick, cohesive biofilm that obstructs the flea’s proventriculus, preventing new blood meals from reaching the midgut [7–9]. Consequently, blocked fleas make repeated attempts to feed, which ultimately leads to the regurgitation of *Y. pestis* into the host.

Although blockage-dependent transmission is considered highly efficient for the spread of *Y. pestis* by fleas [2,4], it may not fully explain all epidemiological patterns. In this context, early-phase transmission may account for rapidly spreading plague epizootics, or for the dissemination of *Y. pestis* by flea species that rarely or never develop proventricular blockage [10–12]. It is worth noting that these two mechanisms are not mutually exclusive but may operate at different stages of flea infection, potentially contributing together to plague persistence and spread.

In Madagascar, plague remains endemic and continues to cause human outbreaks almost every year [1,13], emphasizing the need to better understand the transmission potential of local flea vectors. Several flea species have been identified on the island, including *Xenopsylla brasiliensis* and *Synopsyllus fonquerniei* [14–16]. The former is considered an important vector of *Y. pestis* in East and southern Africa [17–20]. Recent investigations have reported its presence mainly inside houses and an expansion of its geographical range in Madagascar [21], suggesting a potential increase in its public health relevance. In contrast, *S. fonquerniei* is an endemic species widely distributed across Madagascar and mainly found outdoors [14,19,22–24]. Like *X. brasiliensis*, it is suspected to contribute to plague dynamics, as early studies reported its ability to transmit *Y. pestis* from infected to naïve guinea pigs and mice [25,26]. Despite *S. fonquerniei* being confirmed as a plague vector and *X. brasiliensis* suspected to play a role in plague transmission, no data are available on the vector competence or transmission mechanisms [27–32]. This lack of information limits our understanding of their potential contribution to plague epidemiology. Therefore, the primary objective of this study was investigate whether *X. brasiliensis* and *S. fonquerniei* are capable of transmitting *Y. pestis* via early-phase transmission and of developing proventricular blockage under experimental conditions.

## Methods

### Ethics approval and consent to participate

The handling of small mammals followed guidelines established and approved by the Comité d’éthique animale of the Institut Pasteur de Madagascar (Avis n°425/2021/IPM/DS/CEA du 2 Novembre 2021).

### Biological materials

*Xenopsylla brasiliensis* and *S. fonquerniei* populations were used. Fleas were reared in the insectary of the Medical Entomology Unit of the Institut Pasteur de Madagascar, under controlled conditions: 25 ± 2°C and 75% ± 5% relative humidity for *X. brasiliensis,* and 21°C and 80% relative humidity for *S. fonquerniei*. Heparinized mouse blood containing an avirulent *Y. pestis* strain CO92 (lacking the pYV virulence plasmid) was used to infect fleas, and mouse skin served as the natural membrane for the artificial feeding system. Such *Y. pestis* strains lacking the pYV plasmid are commonly used in flea transmission studies, as they retain the ability of *Y. pestis* to colonize the flea digestive tract, and support both flea blockage and EPT [4,33–36]. All animal procedures were approved by the Animal Ethics Committee of the Institut Pasteur de Madagascar (n°425/2021/IPM/DS/CEA, November 2021).

### Flea infection

Flea infections were performed as previously described with slight modifications [8,37]. Two days before infection, a frozen stock of *Y. pestis* was grown in 8 mL of Brain Heart Infusion (BHI) broth at 28°C for 24 h. The culture was then transferred into 92 mL of fresh BHI and incubated at 37°C for 18 h. The optical density (OD₆₀₀) was measured using a Biomate 3S spectrophotometer (Thermo Scientific) to prepare a bacterial suspension adjusted to 5 x 10⁸ bacteria/mL in 1X sterile phosphate-buffered saline (PBS). The bacterial culture was centrifuged at 4400 rpm for 7 min, and the pellet was resuspended in sterile 1X PBS. One milliliter of this bacterial suspension was added to 4.5 mL of heparinized mouse blood to prepare the infectious blood meal.

Prior to infection, fleas were starved for six days. On day 0 (D0), the artificial feeder (50 mm glass feeder, Chemglass Life Science, USA) was assembled with a mouse skin membrane. Infected blood was added to the feeder, then a capsule containing a cohort of approximately 300–600 starved fleas (anesthetized at 4°C) was fixed onto the feeder. The feeder was connected to a circulating water bath (Fisher Scientific) to maintain the blood at 37°C. Fleas were allowed to feed for 1 h 15 min in darkness. Batches of 30 fed fleas were then anesthetized at 4°C and observed under a stereomicroscope to select fed fleas. Approximately 200 fleas (sex ratio: 1:1) were placed in square-bottom *Drosophila* bottles (Genesee Scientific, USA) for subsequent early-phase transmission (EPT) assessment. In addition, 20 fed females were stored at -80°C for later determination of the bacterial load (Colony forming units, CFU) on D0.

### EPT assessment

EPT was assessed from day 1 to day 4 post-infection (p.i.) as previously described [11,37]. Five batches of 10 infected fleas (sex ratio: 1:1) were allowed to feed on sterile mouse blood as described above. The use of flea pools allowed detection of rare transmission events at the batch level, but did not permit individual-level inference. Immediately after feeding, the blood was collected and spread onto blood agar (BA) plates. The mouse skin and glass feeder were washed two to three times with 500 µL of sterile 1X PBS, and the washes were plated on BA. Plates were incubated at 28°C for 48 h, and CFU were enumerated to determine the number of transmitted bacteria. After each feeding, fleas were examined under a stereomicroscope to assess their feeding status and the presence of proventricular blockage. Fed or blocked fleas were individually stored at -80°C for later CFU determination. Between feedings, fleas were maintained under the same insectary conditions described above.

### Determination of CFU in fleas

Bacterial loads were determined as previously described [37]. Frozen fleas were surface-sterilized by sequential immersion in 1 mL of hydrogen peroxide and 1 mL of absolute ethanol for 2 min each, then washed in 10 mL of sterile 1X PBS. Each flea was placed in a 2 mL tube containing 1 mL of PBS and homogenized using a Tissuelyser II (Qiagen, Germany) at 30 Hz. Twenty-five microliters of the homogenate were plated on Lysogeny Broth (LB) agar supplemented with hemin and irgasan (0.1%). CFU were counted after 48 h incubation at 28°C.

### Statistical analysis

Statistical analyses were conducted using R v4.2.2 software [38] and RStudio 2022.07.1 [39]. To compare feeding successes, a Kruskal-Wallis test was used [40]; when significant, Dunn test with Holm correction was applied to perform post-hoc pairwise comparisons [41]. A logistic regression model was chosen to assess the association between transmission and either bacterial loads in fleas (log10) or the number of infected fleas per batch. Transmission efficiency was determined using Maximum Likelihood Estimation (MLE) based on the number of infected fleas that fed on an individual sterile blood device and whether transmission was observed [42]. A significance threshold of P ≤ 0.05 was adopted.

## Results

*Xenopsylla brasiliensis* and *S. fonquerniei* were subjected to two independent infection and transmission assays to assess (i) infection success and bacterial persistence, and (ii) the capacity to transmit *Y. pestis* by EPT. Regardless of the flea population used, the mean feeding success (proportion of blood-fed fleas per batch) ranged between 6% and 54% across sessions and days p.i. (Table 1). For *X. brasiliensis*, feeding success did not differ significantly among days 1–4 during either session 1 (Kruskal-Wallis H-test, *H* = 2.34, *df* = 3, P = 0.51) or session 2 (Kruskal-Wallis H-test, *H* = 4.15, *df* = 3, P = 0.25). For *S. fonquerniei,* feeding success did not differ significantly among days 1–4 during session 2 (Kruskal-Wallis H-test, *H* = 1.04, *df* = 3, P = 0.79). In contrast, during session 1, feeding success differed significantly (Kruskal-Wallis H-test, *H* = 7.84, *df* = 3, P = 0.05), but no significant pairwise differences were detected (Dunn’s post-hoc test with Holm correction, all P > 0.05).

**Table 1:** Feeding success of *Xenopsylla brasiliensis* and *Synopsyllus fonquerniei* across experimental sessions and days post-infection.

Species	Session	Days p.i.	Mean feeding success (%)	SD (%)	No. batches^a^
*Xenopsylla brasiliensis*	1	1	24	28.8	5
		2	32	24.9	5
		3	42	19.2	5
		4	22	13.0	5
	2	1	26	18.2	5
		2	18	13.0	5
		3	50	29.2	5
		4	42	34.2	5
*Synopsyllus fonquerniei*	1	1	22	11.0	5
		2	24	15.2	5
		3	40	28.3	5
		4	6	5.5	5
	2	1	48	13.0	5
		2	54	16.7	5
		3	40	25.5	5
		4	44	11.4	5

^a^each batch is composed of 10 fleas.

Across both experimental sessions, *X. brasiliensis* fleas collected immediately after infection contained mean bacterial loads of 8.5 × 10^4^ CFU per flea (session 1) and 1.5 x 10^4^ CFU per flea (session 2). Most batches had at least one infected flea, with bacterial loads ranging from 0 to 3 x 10^5^ CFU per flea (Tables 2 and 3). These results confirm efficient infection, with some fleas maintaining detectable *Y. pestis* while others appeared to have cleared the bacteria during the days following infection. Transmission of *Y. pestis* was detected during days 1 – 2 p.i and remained infrequent across batches. The number of bacteria transmitted per positive batch was low, ranging from 3 to 6 CFU. Three blocked fleas at day 4 p.i., and none were associated with detectable transmission of *Y. pestis* (Tables 2 and 3). Logistic regression analyses indicated that transmission events were not significantly associated with bacterial load (*β* = -0.13, P = 0.74) or with the number of infected fleas per batch (*β* = -0.75, P = 0.24).

**Table 2:** Early-phase transmission results of *Yersinia pestis* by *Xenopsylla brasiliensis* (session 1).

Days p.i.	Batch	No. of infected fleas and fed fleas^a^	Median CFU per flea and range		No. of blocked fleas (CFU/ blocked flea)	No. of bacteria transmitted	Transmission efficiency (95% CI)
**1**	1	1 - 1	1.9 x 10^4^	–	0	3	0.20 (0.01-0.72)
	2	0	0	–	0	0	
	3	0	0	–	0	0	
	4	5 - 5	2.9 x 10^4^	1.0 x 10^4^ - 4.4 x 10^5^	0	0	
	5	6 - 6	7.3 x 10^3^	8.8 x 10^2^ - 2.6 x 10^5^	0	0	
**2**	1	4 - 5	3.2 x 10^3^	1.2 x 10^3^ - 5.6 x 10^4^	0	0	0.20 (0.01-0.72)
	2	4 - 5	1.0 x 10^5^	4.5 x 10^3^ - 3.6 x 10^5^	0	0	
	3	4 - 5	4.4 x 10^4^	1.6 x 10^4^ - 1.4 x 10^5^	0	0	
	4	1 - 1	3.2 x 10^2^	–	0	6	
	5	0	0	–	0	0	
**3**	1	3 - 5	4.3 x 10^4^	3.2 x 10^3^ - 4.4 x 10^5^	0	0	0.00 (0.00-0.52)
	2	3 - 3	6.7 x 10^3^	4.5 x 10^3^ - 5.1 x 10^4^	0	0	
	3	4 - 4	1.2 x 10^3^	4.0 x 10^1^ - 4.5 x 10^3^	0	0	
	4	2 - 2	1.1 x 10^5^	1.0 x 10^5^ - 1.1 10^5^	0	0	
	5	6 - 7	5.5 x 10^4^	2.7 x 10^3^ - 2.5 x 10^5^	0	0	
**4**	1	3 - 3	3.4 x 10^5^	8.0 x 10^1^ - 4.4 x 10^5^	0	0	0.00 (0.00-0.52)
	2	4 - 4	4.1 x 10^3^	1.6 x 10^2^ - 1.9 x 10^4^	1 (3.0 x 10^5^)	0	
	3	2 - 2	2.8 x 10^4^	1.3 x 10^4^ - 4.3 x 10^4^	1 (2.5 x 10^5^)	0	
	4	1 - 1	1.2 x 10^2^	–	0	0	
	5	1 - 1	2.7 x 10^3^	–	0	0	

^a^each batch is composed of 10 fleas.

**Table 3:** Early-phase transmission results of *Yersinia pestis* by *Xenopsylla brasiliensis* (session 2).

Days p.i.	Batch	No. of infected fleas and fed fleas^a^	Median CFU per flea and range		No. of blocked fleas (CFU/ blocked flea)	No. of bacteria transmitted	Transmission efficiency (95% CI)
**1**	1	2 - 2	3.5 x 10^4^	3.6 x 10^3^ - 3.4 x 10^4^	0	0	0.00 (0.00-0.52)
	2	1 - 1	3.5 x 10^5^	–	0	0	
	3	4 - 4	1.7 x 10^4^	3.2 x 10^2^ - 3.1 x 10^4^	0	0	
	4	1 - 1	8.0 x 10^1^	–	0	0	
	5	5 - 5	4.8 x 10^4^	1.2 x 10^4^–3 x 10^5^	0	0	
**2**	1	3 - 3	1.9 x 10^4^	1.5 x 10^3^ - 3.6 x 10^5^	0	0	0.00 (0.00-0.52)
	2	3 - 3	1.8 x 10^3^	1.2x 10^2^ - 1.6 x 10^5^	0	0	
	3	0 - 0	0	–	0	0	
	4	1 - 1	1.8 x 10^5^	–	0	0	
	5	2 - 2	2.2 x 10^4^	8.6 x 10^3^ - 3.5 x 10^4^	0	0	
**3**	1	6 - 7	2.2 x 10^3^	7.2 x 10^2^- 2.7 x 10^5^	0	0	0.00 (0.00-0.52)
	2	2 - 2	1.3 x 10^4^	9.0 x 10^3^ - 1.6 x 10^4^	0	0	
	3	2 - 3	6.9 x 10^3^	2.8 x 10^2^- 1.4 x 10^4^	0	0	
	4	6 - 9	9.1 x 10^3^	2.8 x 10^2^- 3.2x 10^5^	0	0	
	5	4 - 4	5.1 x 10^3^	4.0 x 10^1^ - 2.6 x 10^5^	0	0	
**4**	1	5 - 7	5.4 x 10^4^	1.2 x 10^3^ - 3.2 x 10^5^	0	0	0.00 (0.00-0.52)
	2	0 - 0	0	–	0	0	
	3	1 - 1	1.2 x 10^2^	–	1 (1.4 x 10^5^)	0	
	4	7 - 9	2.2 x 10^3^	3.2 x 10^2^ - 5.6 x 10^4^	0	0	
	5	4 - 10	2.2 x 10^3^	2.0 x 10^2^- 2.3x 10^4^	0	0	

^a^each batch is composed of 10 fleas.

Under the same experimental conditions, transmission events by *S. fonquerniei* were detected at all tested days p.i. Fleas collected immediately after infection carried mean bacterial loads of 1.4 x 10^3^ (session 1) and 1.0 x 10^4^ CFU per flea (session 2). Furthermore, most batches contained at least one infected flea, with bacterial loads ranging from 0 to 10^5^ CFU per flea (Tables 4 and 5). As observed for *X. brasiliensis*, *Y. pestis* was maintained in some fleas, whereas it appeared to have been cleared in others over time. Compared to *X. brasiliensis*, transmission events were detected at all days p.i., with higher observed frequencies at days 3 and 4 compared with days 1 and 2. A small number of blocked fleas were also observed at days 3 and 4 p.i. (four individuals in total), none of which were associated with detectable transmission (Tables 4 and 5). As for *X. brasiliensis*, transmission was not significantly associated with either bacterial load (*β* = −0.25, P = 0.32) or the number of infected fleas per batch (*β* = −0.29, P = 0.24).

**Table 4:** Early-phase transmission results of *Yersinia pestis* by *Synopsyllus fonquerniei* (session 1).

Days p.i.	Batch	No. of infected fleas and fed fleas^a^	Median CFU per flea and range		No. of blocked fleas (CFU/ blocked flea)	No. of bacteria transmitted	Transmission efficiency (95% CI)
**1**	1	3 - 3	4.8 x 10^4^	2.3 x 10^3^ - 6.5 x 10^4^	0	0	0.40 (0.05-0.85)
	2	3 - 3	2.2 x 10^3^	1.4 x 10^3^ - 2.0 x 10^4^	0	0	
	3	1 - 1	2.9 x 10^3^	–	0	0	
	4	3 - 3	4.8 x 10^2^	2.0 x 10^2^ - 4.1 x 10^3^	0	1	
	5	1 - 1	8.0 x 10^1^	–	0	1	
**2**	1	4 - 5	1.4 x 10^3^	1.6 x 10^2^ - 3.2 x 10^3^	0	0	0.00 (0.00-0.52)
	2	1 - 1	2.0 x 10^3^	–	0	0	
	3	1 - 2	6.8 x 10^2^	–	0	0	
	4	1 - 2	1.2 x 10^2^	–	0	0	
	5	1 - 2	3.2 x 10^2^	–	0	0	
**3**	1	1 - 1	6.8 x 10^2^	–	0	1	0.80 (0.28-0.99)
	2	2 - 6	2.0 x 10^3^	4.0 x 10^2^ - 3.5 x 10^3^	0	1	
	3	3 - 5	1.3 x 10^4^	9.2 x 10^2^ - 1.2 x 10^5^	0	0	
	4	6 - 7	3.8 x 10^2^	4.0 x 10^1^ - 3.1 x 10^3^	0	4	
	5	1 - 1	2.2 x 10^3^	–	0	7	
**4**	1	1 - 1	1.5 x 10^3^	–	0	11	0.60 (0.15-0.95)
	2	0 - 0	0	–	0	0	
	3	1 - 1	3.5 x 10^3^	–	0	4	
	4	1 - 1	1.4 x 10^3^	–	0	1	
	5	0 - 0	0	–	0	0	

^a^each batch is composed of 10 fleas.

**Table 5:** Early-phase transmission results of *Yersinia pestis* by *Synopsyllus fonquerniei* (session 2).

Days p.i.	Batch	No. of infected fleas and fed fleas^a^	Median CFU per flea and range		No. of blocked fleas (CFU/ blocked flea)	No. of bacteria transmitted	Transmission efficiency (95% CI)
**1**	1	4 - 4	2.7 x 10^4^	1.0 x 10^3^ - 8.6 x 10^4^	0	0	0.00 (0.00-0.52)
	2	3 - 4	2.0 x 10^2^	4.0 x 10^1^–2. 0 x 10^2^	0	0	
	3	4 - 4	5.6 x 10^4^	6.0 x 10^2^ - 4.6 x 10^5^	0	0	
	4	4 - 7	1.4 x 10^2^	4.0 x 10^1^ - 4.5 x 10^3^	0	0	
	5	5 - 5	2.7 x 10^4^	1.2 x 10^4^ - 1.4 x 10^5^	0	0	
**2**	1	6 - 8	6.0 x 10^3^	1.2 x 10^2^ - 1.3 x 10^4^	0	0	0.20 (0.01-0.72)
	2	3 - 4	3.6 x 10^4^	5.0 x 10^3^ - 6.0 x 10^4^	0	0	
	3	3 - 6	2.3 x 10^5^	4.0 x 10^4^ - 4.2 x 10^5^	0	0	
	4	3 - 5	1.8 x 10^4^	6.4 x 10^2^ - 7.5 x 10^4^	0	0	
	5	4 - 4	2.4 x 10^4^	1.6 x 10^2^ - 4.7 x 10^4^	0	15	
**3**	1	3 - 6	1.4 x 10^5^	2.1 x 10^3^ - 2.4 x 10^5^	1 (2.4 x 10^4^)	0	0.00 (0.00-0.52)
	2	0 - 0	0	–	0	0	
	3	2 - 3	1.1 x 10^5^	8.9 x 10^4^ - 1.4 x 10^5^	0	0	
	4	5 - 5	4.8 x 10^4^	1.2 x 10^2^ - 2.5 x 10^5^	0	0	
	5	3 - 6	1.2 x 10^5^	8.1 x 10^3^ - 3.7 x 10^5^	0	0	
**4**	1	5 - 6	2.8 x 10^4^	1.2 x 10^2^ - 1.1 x 10^5^	2 (5.2 x 10^2^ - 1.3 x 10^5^)	0	0.00 (0.00-0.52)
	2	4 - 5	7.4 x 10^3^	2.4 x 10^2^ - 3.1 x 10^4^	0	0	
	3	3 - 4	2.8 x 10^4^	2.8 x 10^4^ - 2.8 x 10^5^	0	0	
	4	3 - 4	2.0 x 10^2^	1.2 x 10^2^ - 2.6 x 10^4^	0	0	
	5	3 - 3	5.6 x 10^4^	4.0 x 10^2^ - 1.3 x 10^5^	1 (3.6 x 10^3^)	0	

^a^each batch is composed of 10 fleas.

Together, these results show that both *X. brasiliensis* and *S. fonquerniei* can develop proventricular blockage and support early-phase transmission of *Y. pestis* under experimental conditions, although transmission events were infrequent and not correlated with bacterial load. In *S. fonquerniei* transmission events were more commonly observed at days 3–4 p.i. than at days 1–2.

## Discussion

Madagascar is a plague hotspot with considerable flea diversity [15] including *X. cheopis*, the well-known plague vector. Other species such as *X. brasiliensis* and *S. fonquerniei*, which are also present, remain understudied despite their potential epidemiological importance. Our study is the first to demonstrate that both species can transmit the plague bacillus through the early-phase transmission (EPT) mechanism as early as 24 hours after infection. Such a short latency period may facilitate a rapid pathogen spread and potentially contribute to the early amplification of *Y. pestis*, particularly at the onset of local transmission cycles [11]. In Madagascar, human plague cases are still reported every year [1,13,43–45], reflecting the persistent endemic and epidemic burden of the disease. Although the specific contribution of individual transmission mechanisms cannot be directly inferred from epidemiological data, our findings support the hypothesis that EPT may help explain the rapid onset of transmission observed in some outbreak settings, while its role in field epidemiology remains difficult to quantify. EPT may therefore complement blockage-dependent transmission in shaping plague dynamics.

The ability of both flea species to transmit *Y. pestis* early after infection suggests that they may contribute to transmission dynamics in Madagascar, particularly in areas where they are abundant. Given that *X. brasiliensis* is primarily domestic [16,21], its EPT competence could facilitate transmission to humans, while *S. fonquerniei,* found mostly outdoors, may sustain *Y. pestis* circulation in wildlife reservoirs as previously suggested [22]. These results highlight the need to include *S. fonquerniei* and *X. brasiliensis* in surveillance and vector control strategies, which have so far mainly targeted *X. cheopis* [46–48]. Moreover recognizing the importance of EPT in these species may improve our understanding of *Y. pestis* transmission in plague-endemic regions. Determining their insecticide resistance status will also be essential to guide suitable vector control approaches.

Transmission was independent of the bacterial load or of the number of infected fleas per batch, consistent with previous studies showing that bacterial load does not predict the flea’s ability to transmit *Y. pestis* [12,49–51]. This suggests that EPT is driven by localized and dynamic processes rather than total bacterial burden. Possible explanations include preferential localization of bacteria in the foregut or proventriculus, physiological state or immune responses, which may modulate transmission efficiency [10,33,52]. In particular, transient bacterial adhesion to the esophagus or the entrance of the proventriculus may facilitate regurgitation during the initial phase of blood feeding. Another possible hypothesis is that individual variations in the feeding behavior of fleas could influence transmission outcomes; for instance, some fleas may be more prone to regurgitate at the onset of feeding, thereby increasing the likelihood of transmission. However, this remains hypothetical and requires further research.

Our results showed that batches containing a single infected were frequently associated with transmission events, which is in line with previous findings in *X. cheopis* [11]. While transmission also occurred in batches containing multiple infected fleas, this pattern contrasts with earlier studies in which transmission was generally more frequent in such groups [11,12,50,51]. This observation may reflect individual-level variation or population-specific traits of Malagasy flea populations, but experimental replication will be required to confirm these differences.

Both *X. brasiliensis* and *S. fonquerniei* were competent vectors, yet their transmission patterns differed. *Xenopsylla brasiliensis* transmitted *Y. pestis* only at days 1 and 2 p.i., whereas *S. fonquerniei* transmitted from days 1–4 p.i. These differences further illustrate that vector competence varies between flea species [53]. Similar variability in transmission efficiency and timing has been reported for *Ctenocephalides felis*, *X. cheopis*, and *Oropsylla montana* [11,12,50].

Our work is also the first report of proventricular blockage in *S. fonquerniei* and *X. brasiliensis*, demonstrating their capacity to develop foregut blockage under experimental conditions. Remarkably, blockage was diagnosed earlier (days 3–4 p.i.) than previously reported for other flea species, in which it typically occurs from days 5–31 [10,37,53]. These observations indicate that both species can develop foregut blockage relatively early after infection, although blockage-dependent transmission was not observed under the experimental conditions used here. Differences in the blockage timing could result from species-specific traits, feeding frequency, or anatomical differences at the base of the esophagus-proventriculus junction that deserve further investigation [10,37].

Despite the presence of blocked fleas in our study, no transmission was observed from these individuals. This is consistent with prior studies showing that *C. felis* and *X. cheopis* blocked fleas do not always transmit *Y. pestis* [37,54,55]. Future studies will be needed to assess the role of *S. fonquerniei* and *X. brasiliensis* in plague epidemiology.

The demonstration of early-phase transmission by *X. brasiliensis* and *S. fonquerniei* deepens our understanding of the complexity of plague vector ecology in Madagascar and reveals an additional transmission pathway through which these flea species may contribute to the propagation of *Y. pestis*. The variability in transmission timing and efficiency observed in this study underscores the need for further research into the biological and ecological factors influencing early-phase transmission in these vectors.

## Conclusion

This study demonstrates for the first time that *S. fonquerniei* and *X. brasiliensis* from Madagascar can transmit *Y. pestis* through the early-phase mechanism, as early as 24 hours post-infection. Both species were also capable of developing proventricular blockage within a few days after infection under experimental conditions. Together, these findings suggest that these flea species may contribute to plague transmission dynamics in Madagascar through rapid transmission pathways. Their inclusion in routine surveillance systems and in predictive models of *Y. pestis* transmission may improve the forecasting of plague emergence and spread, and inform vector control strategies in endemic regions.

## References

[1] Bertherat E. Plague around the world in 2019. Wkly Epidemiol Rec. 2019;94:289–92.

[2] Eisen RJ, Gage KL. Transmission of flea-borne zoonotic agents. Annu Rev Entomol. 2012;57:61–82. doi: 10.1146/annurev-ento-120710-100717 21888520

[3] Barbieri R, Signoli M, Chevé D, Costedoat C, Tzortzis S, Aboudharam G, et al. *Yersinia pestis*: The natural history of Plague. Clin Microbiol Rev. 2021;34:1–44. doi: 10.1128/CMR.00044-19 PMC7920731 33298527

[4] Hinnebusch BJ, Jarrett CO, Bland DM. “Fleaing” the Plague: Adaptations of Yersinia pestis to Its Insect Vector That Lead to Transmission. Annu Rev Microbiol. 2017;71:215–32. doi: 10.1146/annurev-micro-090816-093521 28886687

[5] Eisen RJ, Dennis DT, Gage KL. The Role of Early-Phase Transmission in the Spread of Yersinia pestis. J Med Entomol. 2015;52(6):1183–92. doi: 10.1093/jme/tjv128 26336267 PMC4636957

[6] Eisen RJ, Gage KL. Adaptive strategies of Yersinia pestis to persist during inter-epizootic and epizootic periods. Vet Res. 2009;40(2):1. doi: 10.1051/vetres:2008039 18803931 PMC2695026

[7] Hinnebusch BJ, Perry RD, Schwan TG. Role of the Yersinia pestis hemin storage (hms) locus in the transmission of plague by fleas. Science. 1996;273(5273):367–70. doi: 10.1126/science.273.5273.367 8662526

[8] Dewitte A, Bouvenot T, Pierre F, Ricard I, Pradel E, Barois N, et al. A refined model of how Yersinia pestis produces a transmissible infection in its flea vector. PLoS Pathog. 2020;16(4):e1008440. doi: 10.1371/journal.ppat.1008440 32294143 PMC7185726

[9] Bacot AW, Martin CJ. LXVII. Observations on the mechanism of the transmission of plague by fleas. J Hyg (Lond). 1914;13(Suppl):423–39. 20474555 PMC2167459

[10] Hinnebusch BJ, Bland DM, Bosio CF, Jarrett CO. Comparative Ability of Oropsylla montana and Xenopsylla cheopis Fleas to Transmit Yersinia pestis by Two Different Mechanisms. PLoS Negl Trop Dis. 2017;11(1):e0005276. doi: 10.1371/journal.pntd.0005276 28081130 PMC5230758

[11] Eisen RJ, Bearden SW, Wilder AP, Montenieri JA, Antolin MF, Gage KL. Early-phase transmission of Yersinia pestis by unblocked fleas as a mechanism explaining rapidly spreading plague epizootics. Proc Natl Acad Sci U S A. 2006;103(42):15380–5. doi: 10.1073/pnas.0606831103 17032761 PMC1592641

[12] Eisen RJ, Borchert JN, Holmes JL, Amatre G, Van Wyk K, Enscore RE, et al. Early-phase transmission of Yersinia pestis by cat fleas (Ctenocephalides felis) and their potential role as vectors in a plague-endemic region of Uganda. Am J Trop Med Hyg. 2008;78(6):949–56. doi: 10.4269/ajtmh.2008.78.949 18541775

[13] Institut Pasteur de Madagascar. Rapport annuel 2022. Antananarivo: Institut Pasteur de Madagascar. 2023.

[14] Klein JM. Données écologiques et biologiques sur Synopsyllus fonguerniei Wagner et Roubaud, 1932 (Siphonaptera), puce du rat péridomestique, dans la région de Tananarive. Cah l’ORSTOM Ser Entomol Médicale. 1966.

[15] Harimalala M, Ramihangihajason TR, Rakotobe Harimanana R, Girod R, Duchemin J-B. Illustrated Morphological Keys for Fleas (Siphonaptera) in Madagascar. J Med Entomol. 2021;58(4):1701–16. doi: 10.1093/jme/tjab023 33822101

[16] Miarinjara A, Rogier C, Harimalala M, Ramihangihajason TR, Boyer S. Xenopsylla brasiliensis Fleas in Plague Focus Areas, Madagascar. Emerg Infect Dis. 2016;22(12):2207–8. doi: 10.3201/eid2212.160318 27513742 PMC5189135

[17] Kilonzo BS. DDT Resistance in Xenopsylla brasiliensis, the Common Plague Vector in Tanzania. Int J Trop Insect Sci. 1985;6(1):111–4. doi: 10.1017/s1742758400002770

[18] Zimba M, Pfukenyi D, Loveridge J, Mukaratirwa S. Seasonal abundance of plague vector Xenopsylla brasiliensis from rodents captured in three habitat types of periurban suburbs of Harare, Zimbabwe. Vector Borne Zoonotic Dis. 2011;11(8):1187–92. doi: 10.1089/vbz.2010.0095 21142965

[19] Pollitzer R. Plague. Geneve: WHO. 1954. doi: 10.1097/00000441-195502000-00026

[20] Davis DH. Plague in South Africa: a study of the epizootic cycle in gerbils (Tatera brantsi) in the northern Orange Free State. J Hyg (Lond). 1953;51(4):427–49. doi: 10.1017/s0022172400036718 13118145 PMC2217763

[21] Rakotobe Harimanana R, Sebbane F, Rasolofo V, Girod R, Harimalala M. Bioecology of fleas with a focus on the plague vector Xenopsylla Brasiliensis in Mandritsara district, Northern Madagascar. Sci Rep. 2025;15(1):24297. doi: 10.1038/s41598-025-10458-4 40624118 PMC12234880

[22] Harimalala M. Current knowledge on fleas (Siphonaptera) associated with human plague transmission in Madagascar. J Med Entomol. 2025;62(4):749–59. doi: 10.1093/jme/tjaf050 40341394

[23] Beaucournu JC, Randrenjarison AHR, Steven MG. Puces (Insecta: Siphonaptera) d’Ambohitantely, Madagascar: spécificité et phénologie. Malagasy Nat. 2015;9:39–48.

[24] Harimalala M, Rahelinirina S, Girod R. Presence of the Oriental Rat Flea (Siphonaptera: Pulicidae) Infesting an Endemic Mammal and Confirmed Plague Circulation in a Forest Area of Madagascar. J Med Entomol. 2020;57(4):1318–23. doi: 10.1093/jme/tjaa026 32101616

[25] Girard G. Le comportement de la puce Synopsyllus fonquerniei et son role dans la transmission de la peste. Bull la Soc Pathol Exot. 1942;45:189–93.

[26] Brygoo ER, Dodin A. Transmission expérimentale de la peste par Synopsyllus fonquerniei de Madagascar. Comptes rendus Hebd des séances l’académie des Sci Paris. 1967;264:681–2. 4962105

[27] Audoin-Rouzeau F. Chapitre III. Les successeurs: l’étude du bacille et des puces. Les chemins la peste. Rennes: Presses Un. 2003. 375.

[28] Roberts IJ. The transmission of plague in Kenya. J Trop Med Hyg. 1950;53(5):103–9. 24537257

[29] Webster WJ, Chitre DG. Observations of rat-fleas and the transmission of plague. Part IV. Indian J Med Res. 1930;18:407–25.

[30] Coulanges P, Clerc Y, Randrianantoanina E. Etude de X. cheopis et S. fonquerniei, puces pestigènes malgaches - Mise en évidence de leur résistance au DDT, Dieldrin et Malathion. Arch Inst Pasteur Madagascar. 1982;49:171–91. 7186791

[31] Duplantier J-M, Duchemin J-B, Chanteau S, Carniel E. From the recent lessons of the Malagasy foci towards a global understanding of the factors involved in plague reemergence. Vet Res. 2005;36(3):437–53. doi: 10.1051/vetres:2005007 15845233

[32] Chanteau S. Atlas de la peste à Madagascar. Paris: IRD. 2006.

[33] Bland DM, Jarrett CO, Bosio CF, Hinnebusch BJ. Infectious blood source alters early foregut infection and regurgitative transmission of Yersinia pestis by rodent fleas. PLoS Pathog. 2018;14(1):e1006859. doi: 10.1371/journal.ppat.1006859 29357385 PMC5794196

[34] Lemaitre N, Dewitte A, Rakotomanimana F, Gooden D, Toone E, Rajerison M, et al. Assessing the threat of Yersinia pestis harboring a multi-resistant IncC plasmid and the efficacy of an antibiotic targeting LpxC. Antimicrob Agents Chemother. 2025;69. doi: 10.1128/aac.01497-24 PMC11881579 39882860

[35] Hinnebusch BJ, Fischer ER, Schwan TG. Evaluation of the role of the Yersinia pestis plasminogen activator and other plasmid-encoded factors in temperature-dependent blockage of the flea. J Infect Dis. 1998;178(5):1406–15. doi: 10.1086/314456 9780262

[36] Miarinjara A, Bland DM, Belthoff JR, Hinnebusch BJ. Poor vector competence of the human flea, Pulex irritans, to transmit Yersinia pestis. Parasit Vectors. 2021;14(1):317. doi: 10.1186/s13071-021-04805-3 34112224 PMC8194109

[37] Bland DM, Hinnebusch BJ. Feeding Behavior Modulates Biofilm-Mediated Transmission of Yersinia pestis by the Cat Flea, Ctenocephalides felis. PLoS Negl Trop Dis. 2016;10(2):e0004413. doi: 10.1371/journal.pntd.0004413 26829486 PMC4734780

[38] R Core Team. R: A language and environment for statistical computing. Vienna, Austria: R Core Team. 2022.

[39] Posit team. RStudio: Integrated Development Environment for R. http://www.posit.co/ 2025.

[40] Kruskal WH, Wallis WA. Use of Ranks in One-Criterion Variance Analysis. J Am Stat Assoc. 1952;47:583–621. doi: 10.1021/ac00183a752

[41] Dunn OJ. Multiple Comparisons Using Rank Sums. Technometrics. 1964;6(3):241–52. doi: 10.1080/00401706.1964.10490181

[42] Eisen RJ, Holmes JL, Schotthoefer AM, Vetter SM, Montenieri JA, Gage KL. Demonstration of early-phase transmission of Yersinia pestis by the mouse flea, Aetheca wagneri (Siphonaptera: Ceratophylidae), and implications for the role of deer mice as enzootic reservoirs. J Med Entomol. 2008;45(6):1160–4. doi: 10.1603/0022-2585(2008)45[1160:doetoy]2.0.co;2 19058643

[43] Randremanana R, Andrianaivoarimanana V, Nikolay B, Ramasindrazana B, Paireau J, ten Bosch QA. Epidemiological characteristics of an urban plague epidemic in Madagascar, August–November, 2017: an outbreak report. Lancet Infect Dis. 2019;19:537–45. doi: 10.1016/S1473-3099(18)30730-8 30930106 PMC6483974

[44] IPM. Bulletin peste de l’Institut Pasteur de Madagascar. 2024.

[45] IFRC. Disaster relief emergency fund (DREF) Madagascar: Plague outbreak. 2014.

[46] Rahelinirina S, Harimalala M, Rakotoniaina J, Randriamanantsoa MG, Dentinger C, Zohdy S, et al. Tracking of Mammals and Their Fleas for Plague Surveillance in Madagascar, 2018-2019. Am J Trop Med Hyg. 2022;106(6):1601–9. doi: 10.4269/ajtmh.21-0974 35436762 PMC9209941

[47] Miarinjara A, Boyer S. Current Perspectives on Plague Vector Control in Madagascar: Susceptibility Status of Xenopsylla cheopis to 12 Insecticides. PLoS Negl Trop Dis. 2016;10(2):e0004414. doi: 10.1371/journal.pntd.0004414 26844772 PMC4742273

[48] Ratovonjato J. Sensibilité de Xenopsylla cheopis aux insecticides en milieu urbain à Madagascar. Arch Inst Pasteur Madagascar. 1998;64:25–8.

[49] Wilder AP, Eisen RJ, Bearden SW, Montenieri JA, Tripp DW, Brinkerhoff RJ, et al. Transmission efficiency of two flea species (Oropsylla tuberculata cynomuris and Oropsylla hirsuta) involved in plague epizootics among prairie dogs. Ecohealth. 2008;5(2):205–12. doi: 10.1007/s10393-008-0165-1 18787922

[50] Eisen RJ, Wilder AP, Bearden SW, Montenieri JA, Gage KL. Early-phase transmission of Yersinia pestis by unblocked Xenopsylla cheopis (Siphonaptera: Pulicidae) is as efficient as transmission by blocked fleas. J Med Entomol. 2007;44(4):678–82. doi: 10.1603/0022-2585(2007)44[678:etoypb]2.0.co;2 17695025

[51] Eisen RJ, Lowell JL, Montenieri JA, Bearden SW, Gage KL. Temporal dynamics of early-phase transmission of Yersinia pestis by unblocked fleas: secondary infectious feeds prolong efficient transmission by Oropsylla montana (Siphonaptera: Ceratophyllidae). J Med Entomol. 2007;44(4):672–7. doi: 10.1603/0022-2585(2007)44[672:tdoeto]2.0.co;2 17695024

[52] Hinnebusch BJ. The evolution of flea-borne transmission in Yersinia pestis. Curr Issues Mol Biol. 2005;7(2):197–212. doi: 10.21775/cimb.007.197 16053250

[53] Burroughs AL. Sylvatic plague studies: The vector efficiency of nine species of fleas compared with Xenopsylla cheopis. J Hyg (Lond). 1947;45(3):371–96. doi: 10.1017/s0022172400014042 20475778 PMC2234840

[54] Shannon JG, Bosio CF, Hinnebusch BJ. Dermal neutrophil, macrophage and dendritic cell responses to Yersinia pestis transmitted by fleas. PLoS Pathog. 2015;11(3):e1004734. doi: 10.1371/journal.ppat.1004734 25781984 PMC4363629

[55] Lorange EA, Race BL, Sebbane F, Hinnebusch BJ. Poor vector competence of fleas and the evolution of hypervirulence in Yersinia pestis. J Infect Dis. 2005;191(11):1907–12. doi: 10.1086/429931 15871125

